# Detection of *PIK3CA* Mutations in Breast Cancer Bone Metastases

**DOI:** 10.5402/2012/492578

**Published:** 2012-08-30

**Authors:** Manijeh Daneshmand, Jennifer E. L. Hanson, Mitra Nabavi, John F. Hilton, Lisa Vandermeer, Femina Kanji, Susan F. Dent, Mark Clemons, Ian A. J. Lorimer

**Affiliations:** ^1^Centre for Cancer Therapeutics, Ottawa Hospital Research Institute, 501 Smyth Road, Ottawa, Ontario, Canada K1H 8L6; ^2^Department of Pathology, Ottawa Hospital, Ottawa, Canada K1H 8L6; ^3^Department of Medicine, University of Ottawa, Ottawa, Ontario, Canada K1H 8M5; ^4^Department of Biochemistry, Microbiology and Immunology, University of Ottawa, Ottawa, Ontario, Canada K1H 8M5

## Abstract

*Background*. An important goal of personalized cancer therapy is to tailor specific therapies to the mutational profile of individual patients. However, whole genome sequencing studies have shown that the mutational profiles of cancers evolve over time and often differ between primary and metastatic sites. Activating point mutations in the *PIK3CA* gene are common in primary breast cancer tumors, but their presence in breast cancer bone metastases has not been assessed previously. *Results*. Fourteen patients with breast cancer bone metastases were biopsied by three methods: CT-guided bone biopsies; bone marrow trephine biopsies; and bone marrow aspiration. Samples that were positive for cancer cells were obtained from six patients. Three of these patients had detectable *PIK3CA* mutations in bone marrow cancer cells. Primary tumor samples were available for four of the six patients assessed for *PIK3CA* status in their bone metastases. For each of these, the *PIK3CA* mutation status was the same in the primary and metastatic sites. *Conclusions*. *PIK3CA* mutations occur frequently in breast cancer bone metastases. The *PIK3CA* mutation status in bone metastases samples appears to reflect the *PIK3CA* mutation status in the primary tumour. Breast cancer patients with bone metastases may be candidates for treatment with selective *PIK3CA* inhibitors.

## 1. Introduction

An important goal of personalized cancer therapy is to tailor specific therapies to the mutational profile of the individual patient's cancer. A major issue with this strategy is that while the aim of systemic therapy is to treat metastatic disease, typically the primary tumour is used as the source of information on the mutational profile of a patient's cancer. There is clear evidence that tumours evolve over time. For example, Shah et al. used whole genome sequencing to compare the mutation profiles of the primary tumor and a metastatic tumor that occurred many years after the primary and showed that there were multiple additional mutations present in the metastasis [1]. In theory, these differences could arise as a consequence of selection pressures due to therapy or selection pressures for metastatic potential; alternatively, they may arise randomly due to heterogeneity in the primary tumour and/or the high mutation rate in cancer cells. A recent whole genome sequencing study of matched primary and metastatic tumors in pancreatic cancer indicates that both primary tumor heterogeneity and further acquired mutations contribute to differences in mutational profiles between primary and metastatic sites [2].

 The *PIK3CA* gene is frequently mutated in breast cancers as well as other cancers [3]. The product of the *PIK3CA* gene is a catalytic subunit for class IA phosphoinositide 3-kinases (PI 3-kinases). Class IA PI 3-kinases are lipid kinases whose aberrant activation plays a key role in the pathogenesis of many types of cancers [4]. Their aberrant activation occurs by multiple mechanisms including increased activation of upstream tyrosine kinases; loss of the tumour suppressor *PTEN*, which antagonizes PI 3-kinase activity; mutational activation of PI 3-kinase itself. Class IA PI 3-kinases consist of a regulatory subunit and a catalytic subunit. There are three genes encoding different isoforms of the regulatory subunit (*PIK3R1*, *PIK3R2,* and *PIK3R3*) and three genes encoding different isoforms of the catalytic subunit (*PIK3CA*, *PIK3CB,* and *PIK3CD*). In breast cancer, activating point mutations in PI 3-kinase have only been found in the *PIK3CA* gene, although amplification of the *PIK3CB* gene has also been reported in some cases [5]. Within the *PIK3CA* gene, mutational hotspots occur in the regions encoding the helical domain and the kinase domain. The most common helical domain mutations are E542 K and E545 K, while the most common kinase domain mutation is H1047R. Biochemical, tissue culture, and animal studies have confirmed that these mutations are in fact driver mutations [6]. In breast cancer, kinase domain mutations are more common than helical domain mutations [7]. The opposite is true in colorectal cancer; this may relate to the different mechanisms by which the two types of mutations activate PI 3-kinase. *PIK3CA* mutations occur in about 25−30% of breast cancers, with numbers varying depending on the specific patient population and also the types of mutations that are included in the analysis [3]. 

Several studies have evaluated *PIK3CA* mutation status in breast cancer metastases, although none to date have looked specifically in bone metastases [8–10]. Bone is the most frequent site of distant metastasis in breast cancer. Metastatic bone disease is not only incurable, but can also cause significant medical complications including pain, fractures and hypercalcemia. There is therefore an important unmet need to enhance the care of these patients. Here we have assessed the presence of *PIK3CA* mutations in breast cancer bone metastases. We find that these mutations occur frequently in breast cancer bone metastases, suggesting that these patients may be good candidates for treatment with the selective PI 3-kinase inhibitors that are currently under development.

## 2. Materials and Methods

### 2.1. Samples

Ethics approval for this study was obtained from the Ottawa Hospital Research Ethics Board. Samples were obtained from 14 patients at the Ottawa Hospital with histologically confirmed breast cancer and radiological evidence of at least one bone metastasis that was amenable to CT-guided biopsy. Patients underwent an outpatient posterior iliac crest bone marrow aspirate and bone marrow trephine biopsy. In addition, patients underwent an outpatient CT-guided bone biopsy. Details of these procedures have been described previously [11]. For all cases, biopsy samples were formalin-fixed and paraffin-embedded. For a subset of cases where additional biopsy material was obtained, samples were also flash frozen. Sections were cut from formalin-fixed and paraffin-embedded samples after a brief decalcification in which a surface of the block was dipped in decalcification solution. The presence of cancer cells in the biopsies was assessed by a pathologist (M. Nabavi) using hematoxylin and eosin-stained sections. 

### 2.2. Laser Cutting and Laser Capture Microdissection

Isolation of cancer cells from samples was performed with an Arcturus XT instrument (Applied Biosystems Canada, Streetsville, ON, Canada) with both UV laser cutting and laser capture microdissection capabilities. For laser capture microdissection, 5 *μ*m unstained sections from paraffin-embedded samples were mounted on regular glass slides and deparaffinized. Sections were then lightly stained with hematoxylin and dehydrated. Laser capture was done using Arcturus CapSure Macro LCM caps. Cells were captured from a minimum of four slides. For two samples in which the presence of bone fragments in the biopsy would have interfered with laser capture microdissection, sections were mounted on PEN membrane glass slides (Applied Biosystems Canada, Streetsville, ON, Canada) and laser cutting was used to remove bone fragments prior to laser capture. 

### 2.3. DNA Extraction

DNA was extracted using QuickExtract FFPE DNA Extraction Kits (Epicentre Biotechnologies, Madison, WI, USA). Membranes were removed from the laser capture caps and immersed in 25 *μ*L of extraction solution. Samples were then heated for 60 min at 56°C and 2 min at 98°C, as recommended by the manufacturer.

### 2.4. Whole Genome Amplification

Whole genome amplification was performed by the Multiple Displacement Amplification method [12] using DNA REPLI-g Mini Kits (Qiagen, Toronto, ON, Canada). 1.0 *μ*L of nucleic acid extract was used per amplification. Samples were denatured for 3 min at room temperature and neutralized. Amplification was performed for 16 h at 30°C and terminated by heating at 65°C for 3 min. DNA was assayed using Quant-iT PicoGreen dsDNA reagent from Molecular Probes (Eugene, OR, USA).

### 2.5. Positive Control Samples

MCF7 human breast cancer cells (heterozygous exon 9 E545 K mutation) and T47D cells (heterozygous exon 20 H1047R mutation) were from the American Type Culture Collection. Cells were grown in DMEM (MCF7) or RPMI (T47D) media supplemented with 15% fetal calf serum, were passaged for less than six months continuously, and were routinely checked for absence of mycoplasma. Cells growing in 10 cm dishes were washed twice in PBS. 10 mL of 10% neutral-buffered formalin (NBF) was then added. After 2–5 min of incubation, cells were scraped into the NBF and transferred to a 50 mL tube. Cells were left in NBF for a minimum of three hours before further processing. Cells were then washed twice in 80% ethanol and then pelleted in a 1.5 mL microfuge tube with 200 *μ*L of paraffin at the bottom. Pelleted cells were transferred to the cap of a microfuge tube with a spatula and resuspended in 100 *μ*L of 3% low-melting point-agarose. After freezing at −20°C, cells in agarose discs were transferred to cassettes and embedded in paraffin.

### 2.6. PCR and High-Resolution Melt Analyses

The mutation status of exons 9 and 20 in the *PIK3CA* gene was assessed using a nested PCR procedure. The first round of PCR was performed in a multiplexed fashion with outside primers for both exon 9 (forward primer 5′-CTG TGA ATC CAG AGG GGA AA-3′; reverse primer, 5′-GCA CTT ACC TGT GAC TCC ATA GAA-3′) and exon 20 (forward primer 5′-TGA GCA AGA GGC TTT GGA GT-3′; reverse primer, 5′-CCT ATG CAA TCG GTC TTT GC-3′) combined in the same reaction. PCRs were done in a final volume of 60 *μ*L containing 0.1 ng whole genome-amplified DNA, 1.5 mM MgCl_2_, 200 *μ*M dNTPs, 400 nM each primer, and 0.025 units/*μ*L Hotstart Taq polymerase (Qiagen), using an Eppendorf Mastercycler Thermal Cycler. 35 cycles of 95°C for 20 sec, 57°C for 45 sec, and 72°C for 45 sec were run, with an initial denaturation step of 15 min at 95°C and a final extension step of 10 min at 72°C. The second round of the nested PCR and high-resolution melt analysis was performed separately for exon 9 (inside forward primer, 5′-AAG GGA AAA TGA CAA AGA ACA G-3′; inside reverse primer, 5′-CAC TTA CCT GTG ACT CCA TAG AA-3′) and exon 20 (inside forward primer, 5′-GCA AGA GGC TTT GGA TTT C-3′; inside reverse primer, 5′-TTT TCA GTT CAA TGC ATG CTG-3′). PCRs and high resolution melting were done in a final volume of 20 *μ*L containing 0.5 ng first PCR product, 2.5 mM MgCl_2_, 200 *μ*M dNTPs, 200 nM each primer, 5 *μ*M Syto9 dye, and 0.025 units/*μ*L HotStart Taq polymerase, using a Corbett Rotorgene 6000. 30 cycles of 95°C for 20 sec, 57°C for 20 sec and 72°C for 20 sec were run, with an initial denaturation step of 15 min at 95°C. High-resolution melting analysis was performed with an initial hold at 95°C for 1 sec and a melting profile from 72–85°C rising by 0.1 degree each step. Control samples of MCF7 and T47D DNA, prepared as described above, were included in each PCR run.

### 2.7. Sequence Analysis

Template from the initial multiplex PCR (see above) was amplified with M13-tagged primers. PCR products were sequenced directly at StemCore Laboratories, Ottawa Hospital Research Institute, using Big Dye Terminator v 3.1 Chemistry and and an Applied Biosystems 3730 DNA Analyzer.

## 3. Results

### 3.1. Samples

The success rate for performing the three different types of biopsies on the fourteen patients in the study (CT-guided biopsy, trephine bone marrow biopsy, and bone marrow aspiration) is shown in Table 1. Bone marrow aspiration and bone marrow trephine biopsies were performed on 13 of 14 patients, while CT-guided bone biopsies were performed on 9 of 14 patients. A total of 5 of 13 bone marrow trephine biopsies were positive for cancer cells, while 4 of 9 CT-guided bone marrow biopsies were positive and only 2 of 13 bone marrow aspirates were positive. Overall bone metastasis samples positive for cancer cells were obtained from 6 of 14 patients.

### 3.2. Isolation of DNA

A combination of laser cutting and laser capture microdissection was used to isolate >90% pure populations of cancer cells for genomic analyses. As the amount of starting material was small, extracted DNA was subjected to whole genome amplification. Pilot experiments performed in our lab using DNA from formalin-fixed paraffin-embedded pellets of cancer cell lines showed that mutation detection was not affected by the whole genome amplification procedure. DNA suitable for mutation detection was obtained from all samples that were positive for cancer cells, regardless of the type of sampling method used. 

### 3.3. Analysis of *PIK3CA* Mutations

**Figure 1:**
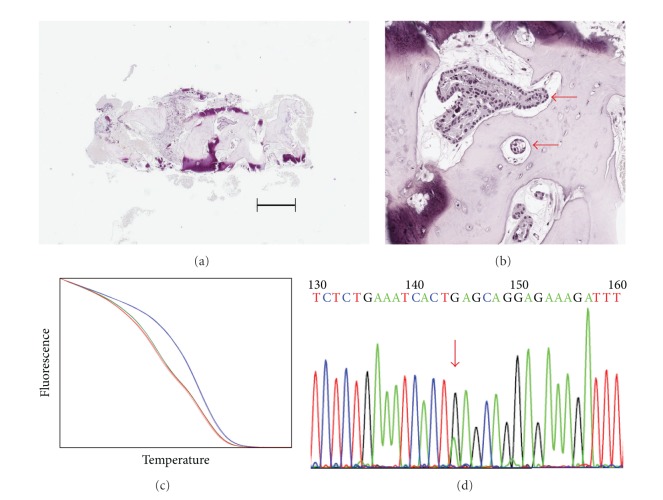
Example of *PIK3CA E545 K* mutation detection from a CT-guided biopsy sample (Case 2).(a) Section from a CT-guided biopsy sample stained with hematoxylin and eosin. The bar shows a distance of 50 *μ*m. (b) Closeup of the same section showing the presence of breast cancer cells (indicated by red arrows). (c) High-resolution melting detection of a heterozygous E545 K mutation in DNA isolated from the sample (blue line, T47D DNA; red line, MCF7 DNA; green line, sample DNA). (d) Confirmation of mutation by Sanger sequencing. The red arrrow indicates the presence of a heterozygous mutation changing the codon GAG to AAG.

**Figure 2:**
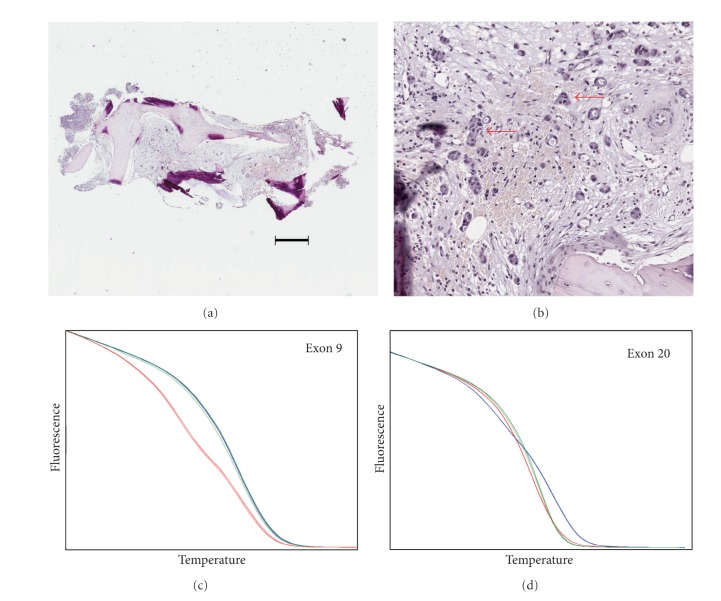
Example of wild-type *PIK3CA* detection from a CT-guided biopsy sample (Case 3).(a) Section from a CT-guided biopsy sample stained with hematoxylin and eosin. The bar shows a distance of 50 *μ*m. (b) Closeup of the same section showing the presence of breast cancer cells (indicated by red arrows). (c) and (d) High-resolution melting shows the absence of mutations in either exon 9 (c) or exon 20 (d) Blue lines, T47D DNA; red lines, MCF7 DNA; green lines, sample DNA. Absence of mutations was confirmed by sequencing.

**Figure 3:**
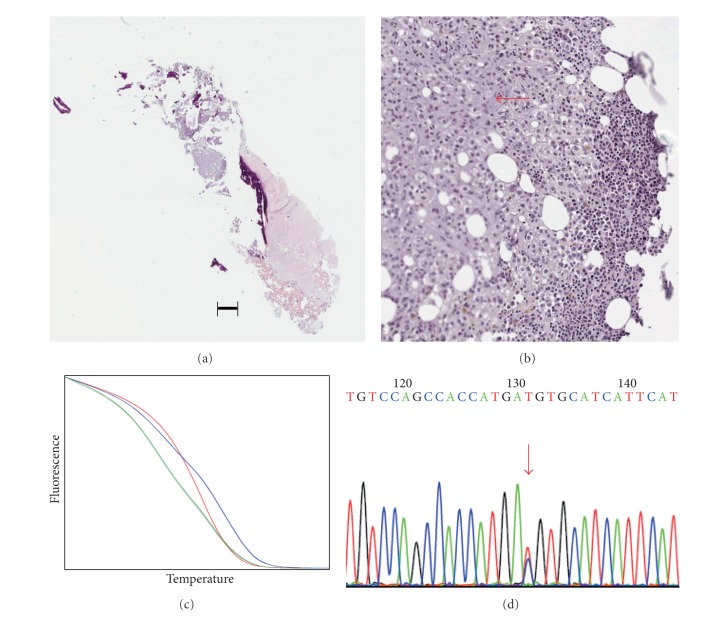
Example of *PIK3CA H1047R* mutation detection from a trephine biopsy (Case 4). (a) Section from a trephine bone marrow biopsy sample stained with hematoxylin and eosin. The bar shows a distance of 50 *μ*m. (b) Closeup of the same section showing the presence of breast cancer cells (region indicated by red arrow). (c) High-resolution melting detection of a heterozygous H1047R mutation in DNA isolated from the sample (blue line, T47D DNA; red line, MCF7 DNA; green line, sample DNA). (d) Confirmation of mutation by Sanger sequencing. The red arrow indicates the presence of a heterozygous mutation changing the codon CAT to CGT (the reverse sequence is shown).

*PIK3CA* mutation analyses were performed blinded to patient information. *PIK3CA* mutations were detected using a nested PCR approach and a high-resolution melting screen as described in Materials and Methods. We restricted our analysis to the three most common mutations, E542 K, E545 K, and H1047R, as these have been well characterized for their ability to constitutively activate *PIK3CA* and function as driver mutations [4]. Dilution with DNA from cultured cells showed that high-resolution melting could detect exon nine E545 K mutations in MCF7 DNA present at 10% of total DNA (a 1 in 20 allele frequency as MCF7 is heterozygous for this mutation). The limit of detection was the same for exon 20 H1047R mutations, assayed using DNA from T47D cells. As the number of samples was small, all were analyzed by Sanger sequencing, regardless of whether or not they showed abnormal melting curves. The two methods for mutation detection showed complete agreement. Of the six cases where biopsies were positive for cancer cells, three showed *PIK3CA* mutations (one heterozygous E545 K mutation, one heterozygous H1047R heterozygous mutation, and one H1047R apparent heterozygous mutation; see Table 1). There was complete concordance in *PIK3CA* mutation status between samples from the same patient taken using the different biopsy methods (Table 1).  Figure 1 shows an example of the detection of an E545 K mutation in a CT-guided biopsy sample; Figure 2 shows an example of detection of wild-type (i.e., nonmutated) *PIK3CA* in a CT-guided biopsy; Figure 3 shows an example of the detection of an H1047R mutation in a trephine bone marrow biopsy sample. 

### 3.4. Analysis of *PIK3CA* Mutations in Primary Tumours

Primary tumor samples were obtained from four of the six cases analyzed for *PIK3CA* bone metastasis mutations. DNA was isolated from these and analyzed as above, except that the whole genome amplification step was omitted. *PIK3CA* mutation analyses in the primary tumor samples were performed blinded to the *PIK3CA* mutation status in the bone metastasis samples. For the four cases, the *PIK3CA* mutation status was the same between the primary and the bone metastasis sample (Table 1).  Table 2 shows the estrogen receptor, progesterone receptor, and *HER2* status of the six patients for which *PIK3CA* mutation status was determined. The elapsed time between patient diagnosis (both cancer diagnosis and metastatic cancer diagnosis) and the bone metastasis biopsy is also shown in Table 2.

## 4. Discussion

In this pilot study, the overall success rate for determining patient *PIK3CA* status in bone metastases was 6/14 (47%). The success rate for patients in which biopsy material contained tumor cells was 100%; thus, the limiting factor in success is the biopsy procedure itself. *PIK3CA *status was assessable from all types of biopsies; future studies should focus on the use of trephine bone marrow biopsies as this procedure had the highest success rate, when both patient willingness to undergo the procedure and the likelihood of obtaining positive samples are taken into consideration. All DNA samples in the study were isolated using laser capture microdissection to avoid loss of sensitivity in mutation detection due to contamination with normal cell DNA. An issue with laser capture microdissection on these samples is that some contain bone fragments that can interfere with the procedure, which relies on close contact between the cap membrane and the tissue section. We found that this problem could be overcome by first using laser cutting to remove bone fragments prior to laser capture microdissection. 

Hotspot *PIK3CA* mutations were found in three of the six assessable samples. While these numbers are clearly too low to draw strong conclusions, this is similar to the expected frequency for a population of ER+ breast cancer patients (all six cases assessed here were ER+). For example, the frequency of *PIK3CA* mutations in a previous study of 366 ER+ breast cancer patients was 38.5% [13]. One patient with H1047R mutation also had *HER2* amplification. Although *PIK3CA* mutations occur at a significantly lower frequency in patients with *HER2* amplification, they do occur in this patient population [13]. Both E545 K helical domain and H1047R kinase domain mutations were detected. With the qualification that the numbers are very small, these data suggest that the overall frequency, types of mutations, and association with other breast cancer biomarkers are similar between breast cancer bone metastases and primary breast cancer tumors.

Recent work has shown that there can be substantial differences in biomarker status between primary breast cancer samples and samples of metastases from the same patient [14]. Of the six patients assessed for bone metastasis *PIK3CA* status in this study, primary tumor material was available for four. There was complete concordance between the *PIK3CA* status in the primary tumor and the bone metastases in these four patients. Other studies have reported conflicting results with regard to concordance in *PIK3CA* mutation status between paired primary and metastatic breast cancer. Jensen et al. reported that in a study of 104 patients, about one-third exhibited differences in their *PIK3CA* mutation status between primary and metastasis samples; it was proposed that this was due to heterogeneity in the primary tumor and selection for the mutation during metastasis [8]. Gonzalez-Angulo et al. reported an 18% discordance rate between paired primary and metastatic breast cancer samples [10]. In contrast, Kalinsky et al. found >90% concordance between primary tumor PIK3CA status and either lymph node or distant metastasis; in addition, they did not find any evidence for heterogeneity in *PIK3CA* status in primary tumors and suggested that the apparent lack of concordance might be due to technical issues [9]. Neither of these studies investigated *PIK3CA* status in bone metastases. Our data indicate that primary tumor *PIK3CA* mutation status (either normal or mutant) is maintained in bone metastases, although a larger number of samples would need to be evaluated in order to determine how invariant this is. Times from initial diagnosis (these correspond to the times when the primary tumor sample was obtained) to the biopsies for this study were performed ranged from <1 to 14 years (Table 2). For three of the four patients in which paired samples were assessed, a period of greater than 10 years elapsed between obtaining the primary tumor sample and the bone metastasis sample. This suggests that *PIK3CA* status does not change substantially over time or in response to standard treatment regimens.

Currently, there are a large number of therapeutic agents under development that target PI 3-kinase. These include dual mTOR/PI 3-kinase inhibitors such as BEZ235 and GSK2126458 [15, 16]; selective class I PI 3-kinase inhibitors such as BKM120 and XL147 [17, 18]; the PI 3-kinase *α* isoform (encoded by *PIK3CA*) selective inhibitor BYL719 (Novartis International AG). In many of the trials with these inhibitors, patients are being preselected for the presence of *PIK3CA* mutations. Our data suggest that these drugs may be active against breast cancer bone metastases and that the *PIK3CA* mutation status in bone metastases can be predicted from readily available primary tumor samples.

## Figures and Tables

**Table 1:** *PIK3CA* mutations in bone metastasis samples.

Case	Bone marrow aspirate	*PI* *K*3*CA* status	Bone marrow trephine biopsy	*PI* *K*3*CA* status	CT-guided bone metastasis biopsy	*PI* *K*3*CA* status	Primary tumour	*PI* *K*3*CA* status
1	neg.	nd	neg.	nd	neg.	nd	no	nd
2	pos.	E545K	pos.	E545K	pos.	E545K	yes	E545K
3	neg.	nd	pos.	WT	pos.	WT	yes	WT
4	neg.	nd	pos.	H1047R	nd	nd	yes	H1047R
5	nd	nd	neg.	nd	neg.	nd	no	nd
6	neg.	nd	neg.	nd	nd	nd	no	nd
7	neg.	nd	neg.	nd	neg.	nd	no	nd
8	neg.	nd	neg.	nd	neg.	nd	no	nd
9	neg.	nd	neg.	nd	neg.	nd	no	nd
10	neg.	nd	neg.	nd	nd	nd	no	nd
11	neg.	nd	pos.	WT	nd	nd	no	nd
12	neg.	nd	nd	nd	pos.	H1047R hom.	no	nd
13	pos.	WT	pos	WT	pos.	neg.	yes	WT
14	neg.	nd	neg.	nd	nd	nd	no	nd

For aspirate, trephine biopsy and CT-guided biopsy, negative (neg.) or positive (pos.), indicate whether or not tumour cells were present in the biopsy. “nd” indicates when a biopsy procedure or an assay was not performed. WT indicates wild-type *PIK*3*CA* sequence at hotspot mutation sites; H1047R and E545K indicate mutations. H1047R hom. indicates that this mutation appeared to be homozygous in this patient (all other mutations were heterozygous).

**Table 2:** Clinical features and pathology of cases with known bone metastasis *PIK3CA* mutation status.

Case		Bone metastasis			Primary			Time from	Time from
	*PIK3CA * status	ER status	PgR status	*HER2* status	ER status	PgR status	*HER2* status	cancer diagnosis (years)	metastatic cancer diagnosis (years)
2	E545K	pos.	pos.	normal	pos.	pos.	normal	11	11
3	WT	pos.	neg.	normal	pos.	neg.	normal	11	1
4	H1047R	pos.	neg.	normal	pos.	pos.	normal	14	2
11	WT	pos.	pos.	normal	pos.	pos.	normal	23	21
12	H1047R hom.	pos.*	neg.*	amplified*	pos.	neg.	amplified	<1	<1
13	WT	pos.	neg.	normal	pos.	pos.	normal	<1	<1

Estrogen (ER) and progesterone (PgR) statuses are shown as positive (pos.) or negative (neg.). Time from cancer diagnosis and metastatic cancer diagnosis is the time between diagnosis and the biopsy taken for this study. *determined from CT-guided bone biopsy; other metastasis receptor and *HER2* data are determined from trephine biopsies.

## Abbreviations

ER: estrogen receptor;
PgR: progesterone receptor;
PI 3-kinase: phosphoinositide 3-kinase;
*PIK3CA:*: gene encoding the class 1A phosphoinositide 3-kinase catalytic subunit isoform;
*PTEN:*: gene encoding a tumor suppressor with lipid phosphatase activity;
*HER2* (official gene name *ERBB2*): gene encoding a member of the epidermal growth factor receptor tyrosine kinase family.
